# Percutaneous Structural Interventions in Adult Congenital Heart Disease: State-of-the-Art Review

**DOI:** 10.14797/mdcvj.1219

**Published:** 2023-05-16

**Authors:** Rody G. Bou-Chaaya, Zhihao Zhu, Valeria E. Duarte, Chun Huie Lin

**Affiliations:** 1 Houston Methodist DeBakey Heart & Vascular Center, Houston, Texas, US; 2 *Rody G. Bou Chaaya and Zhihao Zhu contributed equally

**Keywords:** adult congenital heart disease, structural heart disease, percutaneous interventions

## Abstract

Structural interventions play a crucial role in the management of adult congenital heart disease (ACHD). In recent years, this field has seen significant advancements in catheter-based procedures despite limited investment from industry and lack of device development specific to this population. Because each patient is unique in their anatomy, pathophysiology, and surgical repair, many devices are used off-label with a “best fit” strategy. Therefore, continuous innovation is needed to adapt what is available to ACHD and to increase collaboration with industry and regulatory bodies to develop dedicated equipment. These innovations will further advance the field and offer this growing population less invasive options with fewer complications and faster recovery times. In this article, we summarize some of the contemporary structural interventions performed in adults with congenital defects and present cases performed at Houston Methodist to better illustrate them. We aim to offer a greater understanding of the field and stimulate interest in this rapidly growing specialty.

## Introduction

Over the last decade, significant progress has been made in the diagnosis and management of congenital heart disease (CHD). Improvement in surgical and transcatheter intervention outcomes for children with CHD has led to an increasing number of adults living with CHD.^1^ Currently, the estimated number of patients with adult congenital heart disease (ACHD) is about 2 million individuals in the United States (US). This number is expected to grow in the future as more than 90% of children born with CHD survive into adulthood, specifically those with more complex disease.^2^ As the ACHD population grows, the potential need for transcatheter structural interventions increases. Structural heart disease (SHD) includes a wide range of noncoronary cardiac pathologies, from valve disease and septal defects to baffle leaks and major vessel angioplasty.

Historically, the standard therapy for these types of defects was cardiac surgery. In 1966, Rashkind and Miller performed balloon enlargement of an atrial septal defect (ASD) in three infants to allow intracardiac mixing in the setting of transposition of the great arteries (TGA).^3^ This inspired many invasive cardiologists and paved the way for further interventional intracardiac procedures. Hallmark procedures include transcatheter occlusion of the patent ductus arteriosus by Porstmann et al. in 1967,^4^ transcatheter ASD closure by King et al. in 1976,^5^ and the first percutaneous pulmonary valve replacement by Bonhoeffer et al. in 2000.^6^ The development of new minimally invasive transcatheter techniques and the innovation that followed has led to interventional procedures becoming the primary treatment for many forms of SHD, specifically in the ACHD population.

In this review, we outline several contemporary structural interventions performed in adults with congenital defects (Table 1), summarize these different approaches, and present cases that were completed at Houston Methodist to better illustrate the pathology and the transcatheter intervention.

**Table 1:** List of interventional procedures for adult congenital heart disease.


Closure of patent ductus arteriosus

Closure of atrial septal defects (II ASD and patent foramen ovale)

Closure of acquired and native muscular or perimembranous ventricular septal defects

Embolization of unwanted vessels (coronary fistulas, pulmonary arteriovenous malformations, aortopulmonary collaterals, and venovenous collaterals)

Angioplasty and stenting for coarctation of the aorta

Angioplasty and stenting of right ventricular to pulmonary artery conduit

Angioplasty and stenting of pulmonary artery

Angioplasty and stenting of pulmonary veins

Angioplasty and stenting of Fontan conduit, and fenestrations

Fontan lymphatic interventions

Angioplasty and stenting of atrial switch baffle stenosis, and baffle leak closure

Transcatheter pulmonary valve interventions (balloon valvuloplasty, pulmonary valve replacement, valve-in-valve implantation)

Transcatheter tricuspid valve interventions (valve-in-valve, valve-in-ring, clipping, systemic versus subpulmonic atrioventricular valve)

Closure of paravalvular leaks

Recanalization of obstructed vessels or valves

Left sided interventions (eg, aortic and mitral valve)


## Overview of Achd Transcatheter Interventions

### Closure of Patent Ductus Arteriosus

**Figure 1:**
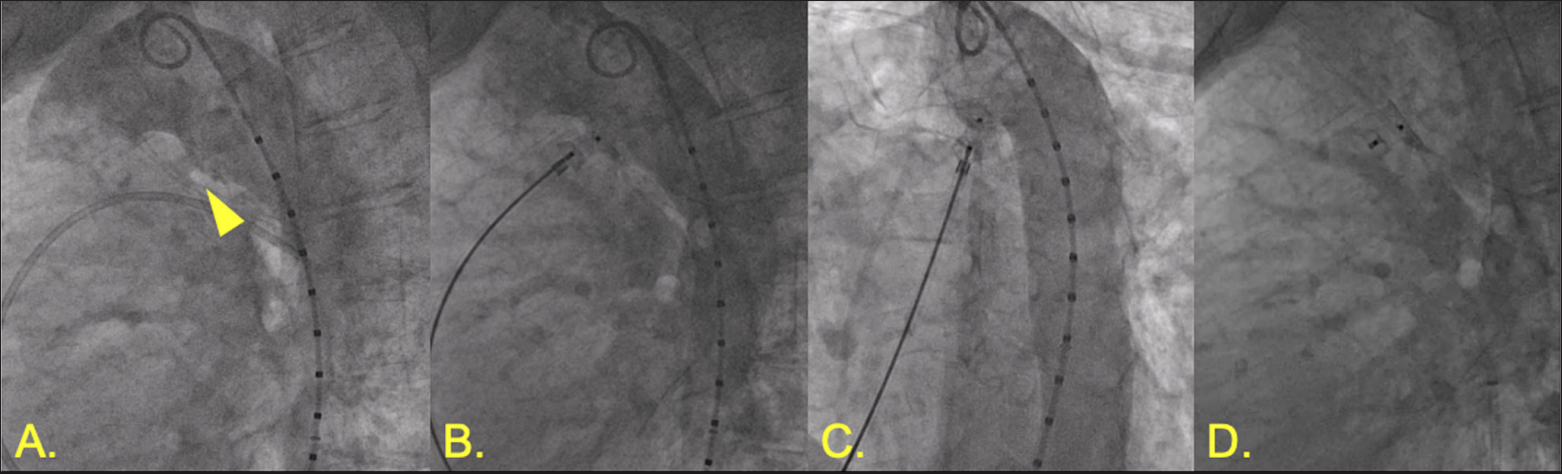
Patent ductus arteriosus (PDA) closure. **(A)** Angiography demonstrating 5.5-mm calcified PDA. **(B,C)** Amplatzer 10-8 Occluder (Abbott) with aortic disc in descending aorta with aortography demonstrating no further shunting. **(D)** Stable position of PDA occluder device following release.

The ductus arteriosus is a fetal vascular connection between the aorta and pulmonary artery that diverts blood away from the pulmonary circulation. Usually, it closes soon after birth. Patent ductus arteriosus (PDA) occurs when this communication persists into adulthood. The incidence reported ranges from 2 to 10 per 10,000 live births, with a well-studied 2:1 female to male predominance.^7,8^ Major clinical manifestations are related to hemodynamic effects from varying degrees of left to right shunting. PDA closure is indicated for hemodynamically significant shunting with signs of volume overload, including left atrial or left ventricular enlargement, as well as pulmonary hypertension. PDA closure is recommended for patients with net left-to-right shunt (Qp:Qs > 1.5) as well as mild to moderately elevated PA pressure and pulmonary vascular resistance (PVR < 5 WU). Transcatheter occlusion is now the preferred method for PDA closure in adults.^9^ Coil embolization with stainless steel or platinum spring coil is the commonly used technique for occlusion of a small PDA. In adults with larger PDA, the most used device is Amplatzer ductal occluder (Abbot Structural Heart) (Figure 1). Multiple multicenter trials have demonstrated high implant success rate with a low rate of major complications using both coil embolization as well as a ductal occluder device.^10,11^

### Closure of Atrial Septal Defects (II ASD and PFO)

Atrial septal defect, one of the most common congenital heart lesions in adults, is often asymptomatic. It can be classified based on location into secundum (70% to 75%): most common, primum, superior sinus venosus, inferior sinus venosus, and unroofed coronary sinus. The primary indication for ASD closure is related to significant left-to-right shunting. In patients with Qp/Qs ≥ 1.5 and without pulmonary hypertension, ASD closure is indicated with or without symptoms. In patients with severe irreversible PAH (PA pressure greater than two-thirds of systemic arterial pressure or PVR greater than two-thirds of SVR) or net right-to-left shunt, the American College of Cardiology/American Heart Association (ACC/AHA) guidelines recommend against ASD closure.^9^ Some evidence also supports additional indications, such as platypnea-orthodeoxia syndrome and paradoxical embolism.^12,13^ Percutaneous closure is the method of choice for secundum ASD.^14^ The two devices approved in the US are the Amplatzer Septal Occluder (Abbot Structural Heart) and Gore CARDIOFORM Septal Occluder/Gore CARDIOFORM ASD Occluder (Gore Medical). Multiple observational studies have shown good efficacy of percutaneous closure similar to surgery but with lower rates of complications and shorter hospital stays.^15,16,17,18,19^ Percutaneous closure has been shown to be associated with improvement to RV and LV function as well as functional capacity.^20^ The most common complications include malposition or embolization of the device and atrial arrhythmias with less common erosion/perforation.^15,17,19^

Sinus venosus ASD is a rare defect (5% to 10% of all ASDs) that occurs at the junction of the superior vena cava and right atrium. It results from a defect in the atrial wall separating the SVC and pulmonary veins and is usually associated with partial anomalous pulmonary venous return of the right pulmonary veins.^21^ These defects have left-to-right shunting at the atrial level but also may have increased shunting due to frequently present and increased pulmonary veinous drainage. They also are at higher risk for developing Eisenmenger syndrome and pulmonary hypertension compared to patients with other ASDs.^22^ The traditional standard of care is surgical correction of the defect. A novel transcatheter technique was first published in 2015, with covered stent correction of the defective posterior wall of the SVC, allowing closure of the sinus venous ASD and redirecting anomalous pulmonary veins to the LA.^23^ Multiple cases and small studies have shown similar techniques as effective alternatives to surgery for patients with suitable anatomy.^24,25,26^ Advanced cross-sectional imaging and 3D modeling for preprocedural planning as well as intraprocedural image guidance have been used with good success for these complex cases.^25,26^

**Figure 2:**
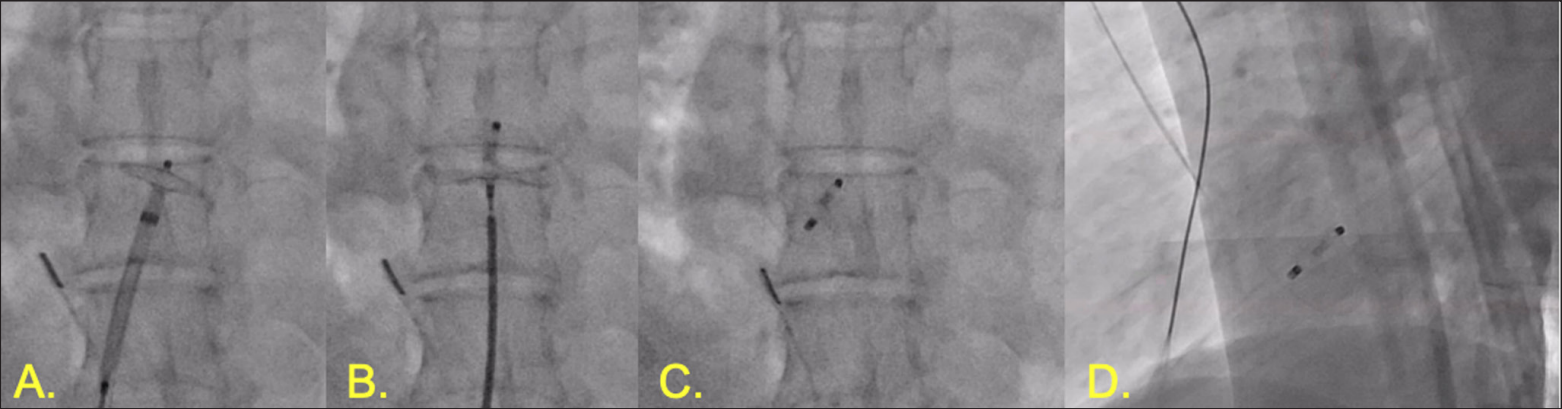
Patent foramen ovale (PFO) occlusion. **(A)** Amplatzer PFO Occluder with left disc deployed in left atrium. **(B)** Right disc deployed against right atrium. **(C)** Amplatzer PFO Occluder deployed. **(D)** Lateral view of Amplatzer PFO Occluder in place.

The foramen ovale is present during fetal development to allow oxygenated blood flow from the right to left atrium. In normal development, the septum primum and septum secundum grow and usually fuse completely by age 2; a patent foramen ovale (PFO) occurs when there is failure of these membranes to fuse. PFO is a relatively common condition, seen in 20% to 30% of the general adult population based on autopsy and echocardiography studies. Most patients with PFO are asymptomatic, but the most common and serious clinical manifestation is paradoxical embolism causing cryptogenic stroke. The REDUCE, RESPECT and CLOSE trials have shown benefit of PFO closure for reducing recurrent ischemic stroke compared with antiplatelet therapy.^27,28,29^ Similar to ASD closure, two devices—the Amplatzer PFO Occluder (Abbot Structural Heart) and Gore CARDIOFORM Septal Occluder—are approved for percutaneous PFO closure in the US (Figure 2).^28^

### Closure of Acquired and Native Muscular or Perimembranous Ventricular Septal Defects

Ventricular septal defects (VSDs) comprise around 10% of patients with ACHD and are mostly diagnosed and treated before adulthood. Spontaneous closure is frequent in childhood. VSD is classified by location as perimembranous/subaortic (80% and most common), muscular, and inlet/outlet. They also can be acquired post-surgical repair as residual-patch VSDs, post-infarction, and post-traumatic.^30^ Closure in adults is recommended for left-to-right shunt greater than 1.5:1 without pulmonary hypertension (PVR < 3 WU), evidence of LV volume overload, or in VSD-associated aortic regurgitation or recurrent endocarditis.

Transcatheter closure using Amplatzer devices has increasingly become an alternative, especially in residual-patch VSD and those located centrally in the interventricular septum. Perimembranous VSD closure is feasible but carries the risk of complete heart block and tricuspid and aortic valve tissue entrapment, leading to regurgitation.^31,32^ Ultimately, a new device design is needed to provide better stability with minimal trauma to the conduction system. Hybrid closure approaches currently exist without the need for cardiopulmonary bypass. They consist of a periventricular occluder device delivery system inserted through the chest and RV wall followed by device closure in the usual fashion.^33^

### Closure Of Aortopulmonary and Veno-Venous Collaterals

Patients with single ventricle physiology survive into adulthood following a palliative Fontan operation.^34^ The Fontan allows direct connection of the systemic venous system to the pulmonary arterial circulation. These patients often develop chronically elevated systemic venous pressures, which result in multiple complications including formation of collaterals. Aortopulmonary collaterals (APC) have a complex effect on hemodynamics and systemic oxygen saturation due to increasing pulmonary blood flow.^35^ Embolization may provide short-term hemodynamic benefit but may worsen systemic desaturation. On the other end, venovenous collaterals (VVC) provide a decompressive effect for Fontan pressures but lead to systemic desaturation. Embolization of VVCs can lead to increased Fontan pressure with increased long-term mortality, especially in those patients with previously elevated Fontan pressures.^34^ Studies have shown that embolization to eliminate APCs and VVCs prior to orthotopic heart transplant and combined heart/liver transplant are associated with decreased bleeding during the transplant operation.^36^

### Angioplasty and Stenting for Coarctation of the Aorta

**Figure 3:**
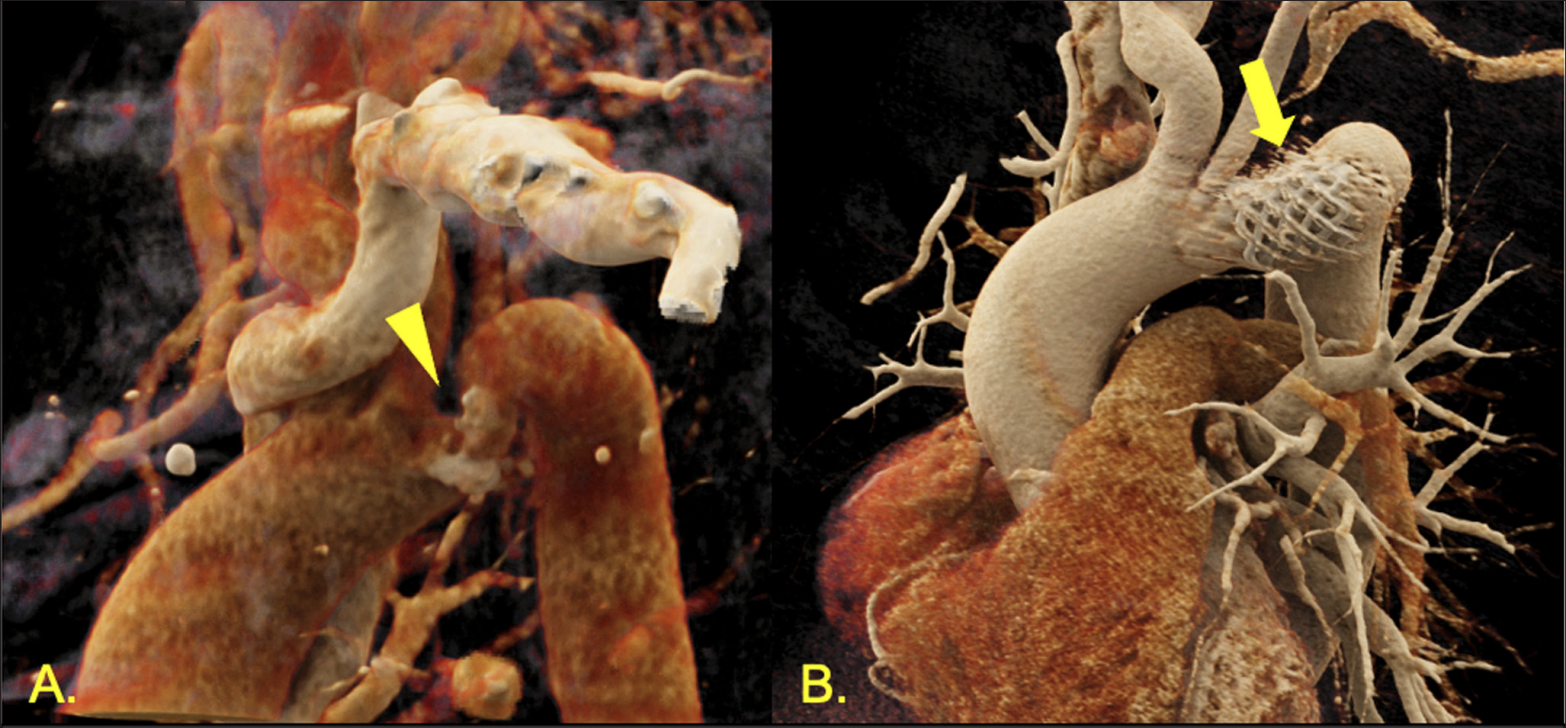
Three-dimensional reconstruction of computed tomography angiograms. **(A)** Coarctation of the aorta at aortic arch preintervention (triangle showing calcified coarctation). **(B)** Postintervention view with covered stent in place (arrow showing stent).

**Figure 4:**
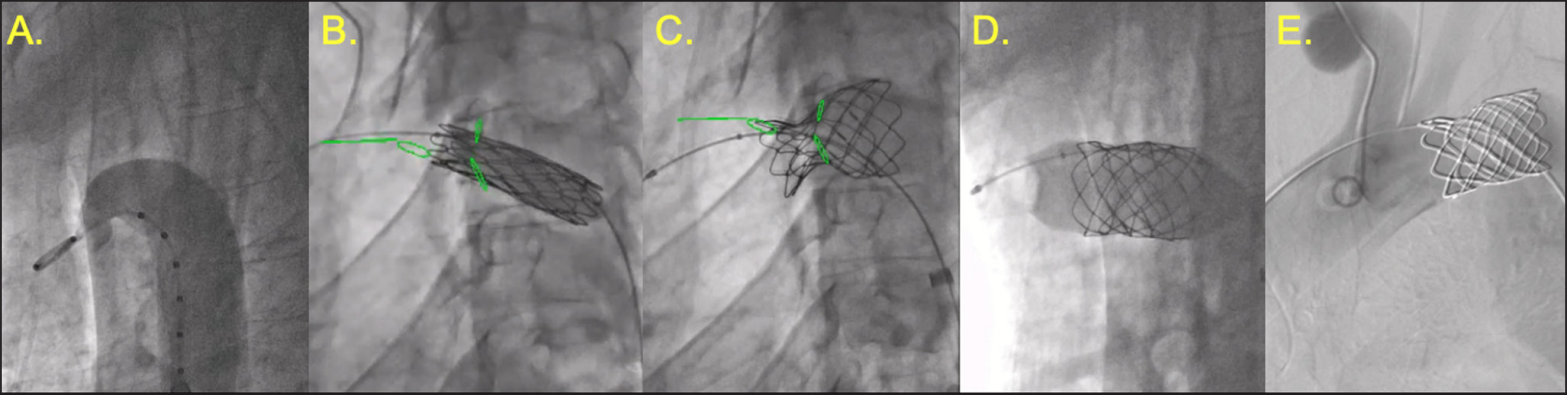
Aortic coarctation angioplasty. **(A)** Pre-stent angiography demonstrating coarctation of aortic arch. **(B)** Expansion of inner balloon during angioplasty with Cheatham platinum covered stent 24 mm × 3 cm. **(C)** Full deployed stent with significant waist due to calcified coarctation. **(D)** 24 × 4 Atlas Gold balloon (Becton, Dickinson and Company) used for post-dilation. **(E)** Post-stent digital subtraction angiography demonstrating patent stent with no residual stenosis, dissection, or extravasation.

Coarctation of the aorta (CoA) is a relatively common congenital defect with an incidence of 4 in 10,000 live births, or 5% to 8% of all congenital heart disease. It is frequently associated with other lesions such as bicuspid aortic valve (> 50%), cerebral “berry” aneurysm, and aortic wall pathology (eg, cystic medial necrosis, aneurysm, and dissection).^37^ A small number of patients remain undiagnosed until adulthood and present with secondary hypertension, headaches, or nosebleeds. The ACC/AHA guidelines recommend intervention for a significant native or recurrent aortic coarctation (upper extremity/lower extremity resting peak-to-peak gradient > 20 mm Hg) and/or if there is radiologic evidence of coarctation and collateral flow.^9^ Open surgical repair and angioplasty with stenting have comparable intraoperative mortality rates of 0% to 3%. The complications of CoA repair include re-coarctation, aneurysm, pseudoaneurysm, and dissection. An observational prospective study has shown that angioplasty with stenting had fewer complications than surgical and balloon angioplasty patients (2.3%, 8.1%, and 9.8%, respectively; *P* < .001) with an aortic wall injury rate of 12.6% in surgical, 7.1% in stent, and 43.6% in balloon angioplasty patients.^38^ Therefore, given the concern for aortic wall injury, the practice has shifted towards the routine use of Covered CP Stents (Braun Interventional Systems Inc.) that were proven to effectively treat and potentially prevent aortic wall injury associated with CoA (US COAST II trial) (Figures 3, 4).^39^ However, it is important to note that some concerns persist regarding re-coarctation and stent fracture risk over time despite the protection that the Covered CP Stent is purported to provide.^40^

### Angioplasty and Stenting of Right Ventricular to Pulmonary Artery Conduit

**Figure 5:**
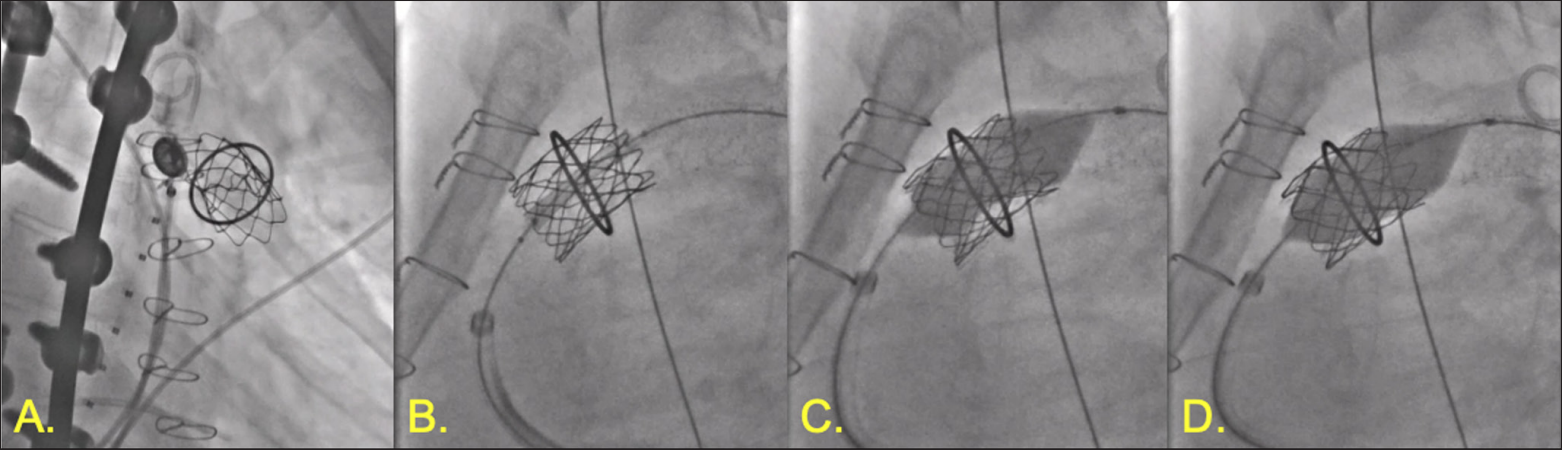
Right ventricular outflow tract and valvuloplasty. **(A,B)** Hancock bioprosthetic valve (Medtronic) with previous Melody valve (Medtronic). **(C)** Valvuloplasty and deployment of Palmaz 3110 XL stent (Cordis) with NuMED balloon-in-balloon device (NUMED, INC). **(D)** Post-dilation using Atlas Gold balloon {Becton, Dickinson and Company).

Right ventricular outflow tract obstruction is associated with multiple complex congenital defects such as pulmonary atresia, tetralogy of Fallot, common arterial trunk, and Ross and Rastelli procedures. When the native outflow tract is not amenable to reconstruction, surgical conduits are inserted to allow continuity between the RV and the PA. Despite newer surgical techniques and conduits, more than 60% of conduits require reoperation at 20 years, with conduit stenosis being the most common reason.^41^ To decrease the need for repeat sternotomies, angioplasty and stenting is used as an alternative to prolong the conduit lifespan. According to a study that included 221 patients with obstructed RV-PA conduits, the median freedom from conduit surgery after stenting was 3.9 years in patients > 5 years post-procedure. Although stent fractures were common, they were not associated with hemodynamic compromise or shorter time to conduit surgery.^42^ In case significant conduit regurgitation exists with the RV-PA conduit stenosis, stenting alone, transcatheter pulmonary valve replacement, or a replacement with a valved RV-PA conduit should be considered on a case by case basis depending on the size of the conduit, valve competence, and risk of infection (Figure 5).

### Angioplasty and Stenting of Pulmonary Artery

Pulmonary artery stenosis is a congenital or acquired disease that can affect different sites of the pulmonary artery and its branches. The location and etiology of stenosis are key factors in choosing the angioplasty modality. Simple high-pressure balloon angioplasty (BA) is usually used for compliant and proximally located vessels with reasonable success rates of about 60% to 72% in congenital pulmonary artery stenosis.^43^ Cutting BA is utilized for dilation-resistant PA and distal branch stenosis. Stent placement is the most effective modality but is reserved for lesions that fail BA, given the need for serial dilations needed to match somatic growth. Currently, new bioresorbable stents are being studied to avoid re-interventions.^44^

### Angioplasty and Stenting of Pulmonary Veins

**Figure 6:**
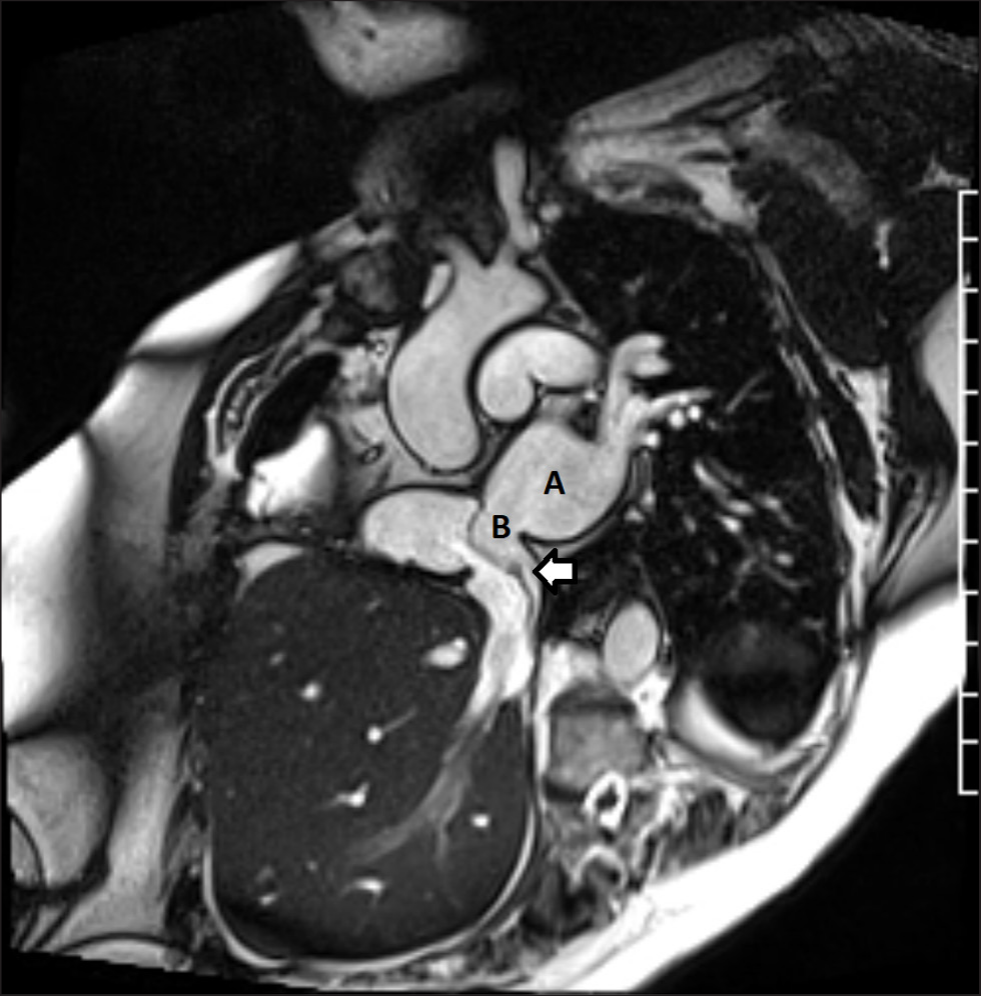
Cardiac magnetic resonance imaging post scimitar repair showing the scimitar vein baffle draining into the left atrium through a surgically created atrial septal defect (ASD). **(A)** Left atrium; **(B)** surgical ASD. Arrow: scimitar vein baffle

**Figure 7:**
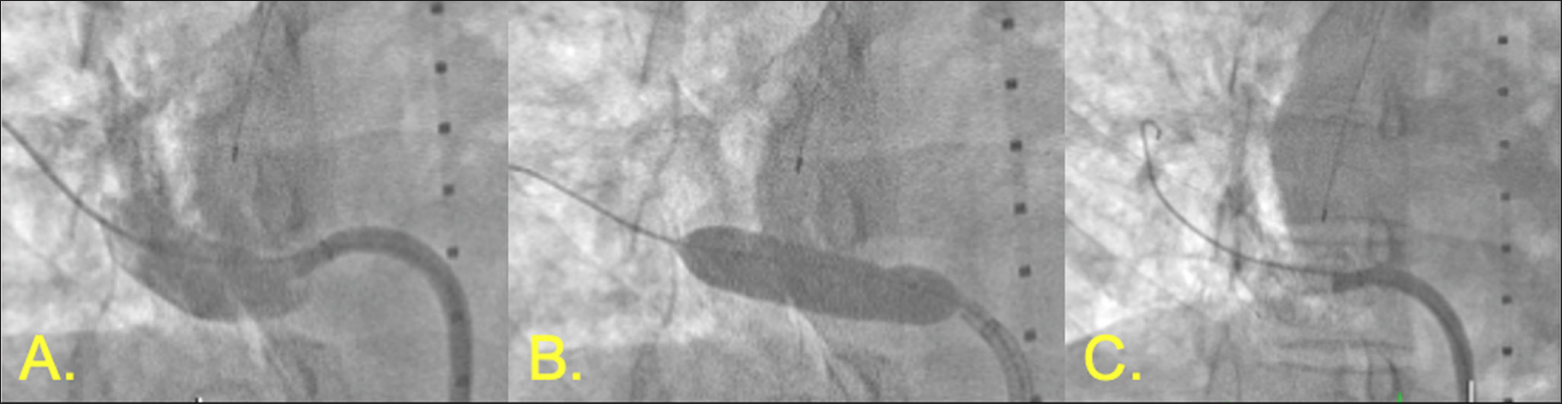
Intervention for stenosis of reattached right pulmonary vein in scimitar syndrome. **(A)** Venography demonstrating stenotic right pulmonary vein. **(B)** Inflation of Medtronic drug-eluting balloon. **(C)** Post-intervention with Genesis Palmaz 29- × 10-mm stent (Cordis).

Pulmonary vein stenosis (PVS) is a relatively rare anomaly that can be either primary or acquired throughout life. In its isolated form, congenital PVS is usually seen almost exclusively in young children. However, acquired PVS can be seen in both children and adults. It results from mediastinal processes such as neoplasms or fibrosing mediastinitis, pulmonary vein isolation during atrial fibrillation ablation, and surgical correction of anomalous pulmonary venous return (ie, Scimitar syndrome; Figures 6, 7). In infants, surgery is preferred, and stenting the pulmonary veins should be reserved as a last resort before lung transplantation given the high incidence of restenosis and the need for repeat dilation of the stent as the child grows.^45^ In adults, BA and stenting are both effective therapeutic options. However, restenosis remains a concern, especially in the BA group where a recent meta-analysis has shown lower restenosis and reintervention rates with stent placement.^46^

### Angioplasty and Stenting of Fontan Conduit, Fenestration Closure, and Fontan Lymphatic Interventions

The Fontan procedure is the last step of multiple operations used to treat congenital cardiac anomalies with single ventricle physiology. A total cavopulmonary connection is created by using extracardiac conduits (ECC) to anastomose the systemic veins to the pulmonary arteries.^47^ However, about 14% to 18% of Fontan ECC are prone to stenosis over time, which leads to progressive venous congestion, reduced exercise tolerance, and ultimately Fontan failure.^48^ The indication for angioplasty is a reduction in the size of the conduit (~25%) rather than a gradient since a gradient is not always present.^9^ Angioplasty with stenting is preferred to treat isolated conduit stenosis. Multiple stents have been used, with covered stents advised in the treatment of highly calcified conduits to prevent conduit dissection. Success rates are close to 100%, with a complication rate around 0% to 10%. The need for re-dilation was seen in younger patients with growth potential.^49^

**Figure 8:**
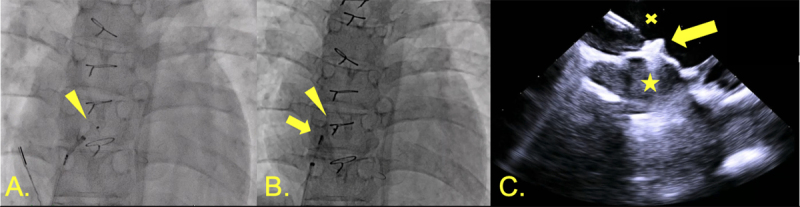
Fontan fenestration closure. **(A)** Left disc of 4-mm Amplatzer ventricular septal defect closure device deployed in common atrium (triangle). **(B)** Right disc now also deployed in Fontan (arrow). **(C)** Intracardiac echocardiography showing device deployment (arrow) across fenestration from common atrium (star) to Fontan (x).

Good Fontan hemodynamics are defined by a broad connection, with low pulmonary vascular resistance leading to less congestion with improved cardiac output. Sometimes, especially in the postoperative phase, surgeons create a fenestration between the ECC and the systemic atrium to avoid extreme congestion and low cardiac output. However, as the size of the fenestration increases, the oxygen saturation decreases, and a balance is needed. Percutaneous options are therefore available to create a fenestration, enlarge it, or close it, depending on the clinical need (Figure 8). Persistence of a fenestration can result in cyanosis and limited exercise tolerance. This can be palliated by fenestration closure using devices such as Amplatzer Atrial Septal Occluder (4-6 mm), Amplatzer duct occluder II, or other vascular plugs.^50^

Chronically elevated systemic venous pressure impairs effective drainage of the lymphatic system. Two major complications: protein-losing enteropathy and plastic bronchitis are directly related to lymphatic dysfunction in failing Fontan circulation. A small case series describes the embolization of dilated lymphatics in the gut and in branches of the thoracic duct with improvement of symptoms after limited follow up.^51,52^

### Angioplasty and Stenting of Atrial Switch Baffle Stenosis and Baffle Leak Closure

Transposition of the great arteries is characterized by atrioventricular concordance and ventriculoarterial discordance. In the 20th century, atrial switch operations (Mustard and Senning) were performed. Atrial venous tunnels (baffles) were used to reorient the systemic and pulmonary venous return to the opposite ventricle. These procedures improved long-term survival from 71% to 94% at 20 years of age.^53^ However, baffle stenosis or leaks remain common complications, leading to decreased exercise capacity, paradoxical emboli with left-to-right shunting, and reintervention.^54^ Technically feasible baffle interventions are recommended for symptomatic patients and in asymptomatic ones with substantial ventricular overload. Multiple case series have shown that percutaneous baffle stenting and baffle leak closure is a safe and effective alternative to surgery in this population.^55^

### Transcatheter Pulmonary Valve Interventions

Balloon valvuloplasty remains the mainstay of therapy for congenital pulmonary valve stenosis with excellent long-term outcomes in all age groups. The results are comparable to surgical valvotomy, with low recurrence rates.^56^

**Figure 9:**
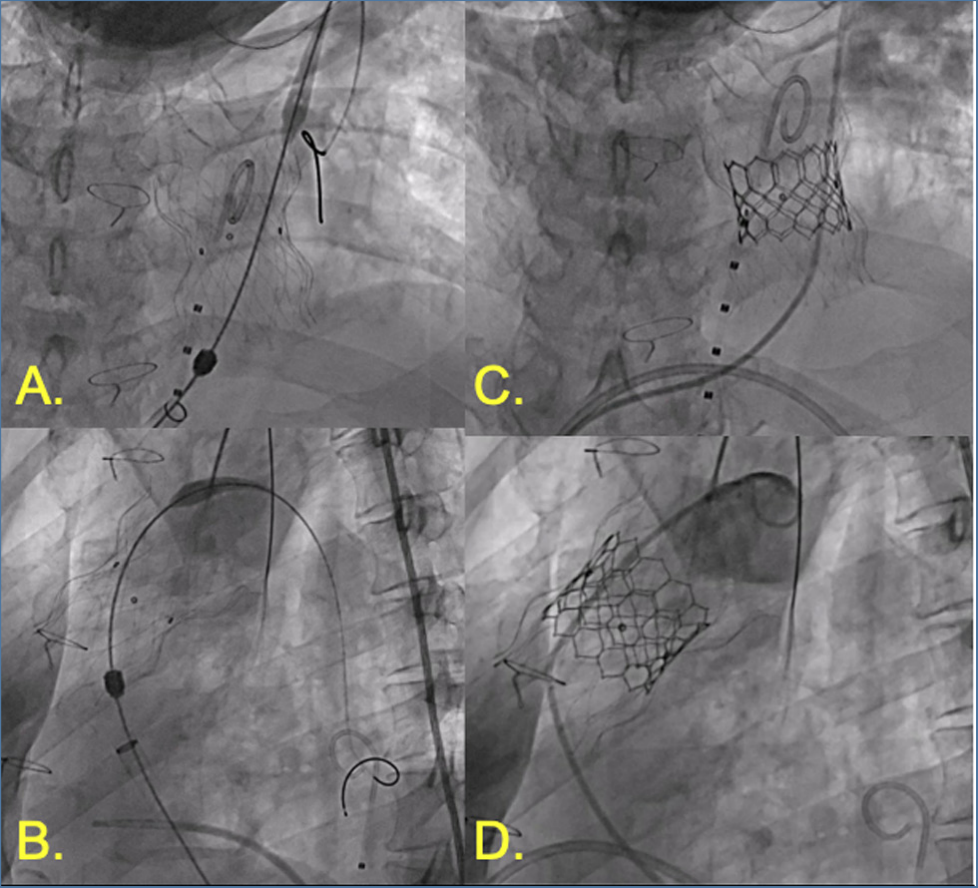
Percutaneous pulmonary valve replacement. **(A)** Anterior-posterior view of Edwards Alterra pre-stent in right ventricular outflow tract. **(B)** Lateral view of Alterra. **(C)** AP view of Edwards SAPIEN 3 valve deployed in Alterra. **(D)** Lateral view of SAPIEN 3 valve in position.

Transcatheter pulmonary valve replacement (tPVR) is one of the recently developed interventions in ACHD. It has quickly evolved to become the treatment of choice in patients with suitable right ventricular outflow tract (RVOT) morphology.^57^ The two major adult groups requiring tPVR are (1) Tetralogy of Fallot (ToF) patients with right ventricle-pulmonary artery (RV-PA) valved conduits or surgical bioprosthetic valves as part of ToF repair during childhood, and (2) patients with congenital aortic stenosis that underwent a Ross procedure, where the pulmonary valve is autotransplanted to replace the aortic valve and an RV-PA conduit is used. Two major clinical trials that evaluated the dominant balloon expandable valve systems (Melody and SAPIEN) showed excellent results in restoration of valve function, New York Heart Association functional class improvement, and approximately 98% freedom from all-cause mortality at 3 years.^58,59^ However, roughly 75% of patients with dilated or patched native RVOT are not candidates for balloon-expandable valve systems. Therefore, self-expanding systems (Harmony, Venus P Valve, Alterra Pre-Stent/SAPIEN, and Pulsta valves) have been increasingly used as an alternative to hybrid plication of the RVOT for balloon-expandable valve systems (Figure 9, Videos 1, 2, 3, 4, 5). As this procedure grows, it is important to recognize associated complications, such as compression of the coronary arteries, aortopulmonary fistulae, and endocarditis with rates of up to 8% with the Melody valve.^60^

**Video 1:** Parasternal right ventricular outflow view with color Doppler showing moderate to severe pulmonary regurgitation prior to Edwards SAPIEN 3 transcatheter pulmonary valve system with Alterra adaptive pre-stent; see also at https://youtu.be/QN5lkKBQzrM.

**Video 2:** Parasternal right ventricular outflow view with color Doppler showing successful placement of Edwards SAPIEN 3 transcatheter pulmonary valve system with Alterra adaptive pre-stent. Trivial paravalvular leak is noted; see also at https://youtu.be/4IdjE0wN6a0.

**Video 3:** Fluoroscopy showing the Alterra adaptive pre-stent delivery system in the right ventricular outflow tract with deployment of the distal portion of the Alterra; see also at https://youtube.com/shorts/O-MEnsP9ZFc.

**Video 4:** Fluoroscopy showing complete deployment of the self-expanding Alterra adaptive pre-stent in the right ventricular outflow tract; see also at https://youtube.com/shorts/Hb64ywc_rsU.

**Video 5:** Fluoroscopy showing the SAPIEN 3 transcatheter pulmonary valve being implanted within the Alterra adaptive pre-stent; see also at https://youtube.com/shorts/iTxb0ymN6Vw.

### Transcatheter Tricuspid Valve Interventions

**Figure 10:**
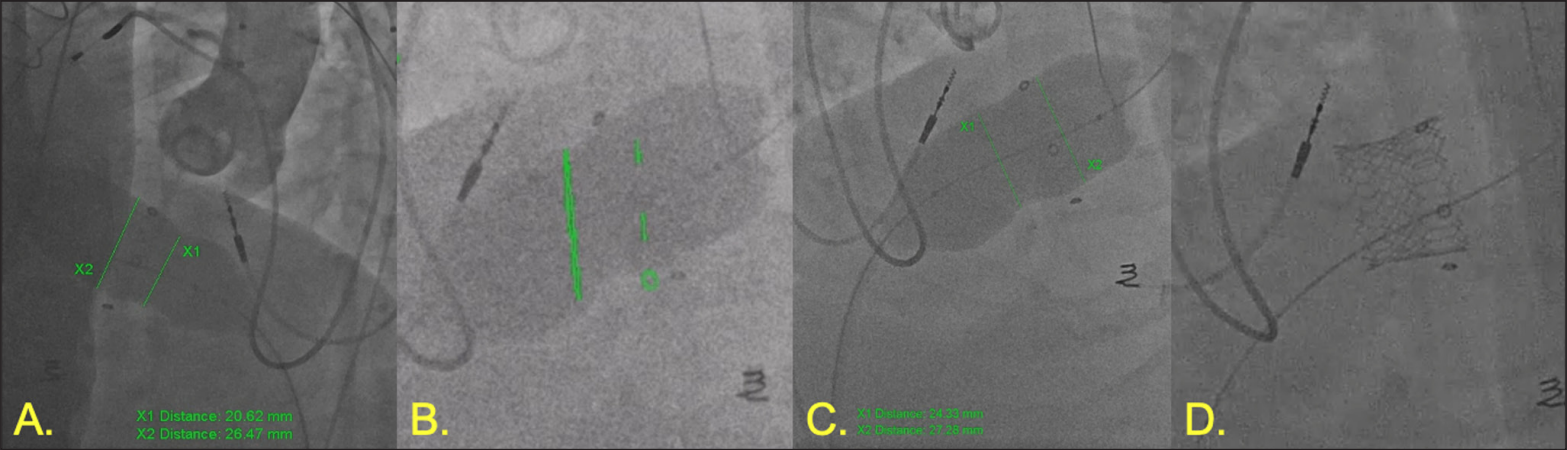
Bioprosthetic tricuspid valvuloplasty. **(A)** 26-mm Vida valvuloplasty balloon inflated showing 20.6 mm at waist. **(B)** 28-mm True balloon inflated for intentional value fracture (green markers showing landmarks created using 2D/3D registration from computed tomography angiography). **(C)** Post valve fracture measurement with improvement. **(D)** SAPIEN 3 Ultra 26-mm valve successfully deployed.

Interventions including valve-in-valve, valve-in-ring, clipping, and systemic versus subpulmonic atrioventricular valves are discussed in the transcatheter tricuspid valve interventions section of this issue. However, we report the case of a patient with a history of a redo surgical bioprosthetic 27 mm mosaic valve at age 37. He presented with tricuspid valve stenosis and underwent tricuspid bioprosthetic valvuloplasty with intentional valve fracture and deployment of SAPIEN 3 Ultra 26 mm (Figure 10).

## Conclusion

Interventions for ACHD comprise a wide range of procedures that have grown significantly in recent years. With continued advances in technology, imaging techniques, and understanding of long-term outcomes, the number of transcatheter interventions will continue to rise. However, greater focus on collaboration with industry is required to ease pathways for device development and introduce innovative strategies for minimally invasive therapeutic approaches.

## Key Points

- As the population of patients with adult congenital heart disease (ACHD) grows, the potential need for transcatheter interventions increases.
- ACHD interventions include a wide range of cardiac procedures that extend from shunt closure to major vessel angioplasty and valve replacement.
- Intervening in this population can be challenging because each patient is unique in their anatomy, pathophysiology, and surgical repair.
- A multidisciplinary team approach is of critical importance to offer the best possible outcome.
- Continuous innovation is needed to adapt what is available to ACHD and to increase collaboration with industry and regulatory bodies to develop dedicated equipment.

## Data Accessibility Statement

The original contributions presented in the study are included in the article/supplementary material; further inquiries can be directed to the corresponding authors.
